# Inflammatory Serum Protein Profiling of Patients with Lumbar Radicular Pain One Year after Disc Herniation

**DOI:** 10.1155/2016/3874964

**Published:** 2016-05-11

**Authors:** Aurora Moen, Anne-Li Lind, Måns Thulin, Masood Kamali-Moghaddam, Cecilie Røe, Johannes Gjerstad, Torsten Gordh

**Affiliations:** ^1^National Institute of Occupational Health, 0033 Oslo, Norway; ^2^Department of Physical Medicine and Rehabilitation, Oslo University Hospital, 0424 Oslo, Norway; ^3^Department of Surgical Sciences, Uppsala University, 751 85 Uppsala, Sweden; ^4^Department of Statistics, Uppsala University, 751 20 Uppsala, Sweden; ^5^Department of Immunology, Genetics and Pathology, Science for Life Laboratory, Uppsala University, 751 85 Uppsala, Sweden; ^6^Faculty of Medicine, University of Oslo, 0316 Oslo, Norway; ^7^Department of Molecular Biosciences, University of Oslo, 0371 Oslo, Norway

## Abstract

Earlier studies suggest that lumbar radicular pain following disc herniation may be associated with a local or systemic inflammatory process. In the present study, we investigated the serum inflammatory protein profile of such patients. All 45 patients were recruited from Oslo University Hospital, Ullevål, Norway, during the period 2007–2009. The new multiplex proximity extension assay (PEA) technology was used to analyze the levels of 92 proteins. Interestingly, the present data showed that patients with radicular pain 12 months after disc herniation may be different from other patients with regard to many measurable serum cytokines. Given a false discovery rate (FDR) of 0.10 and 0.05, we identified 41 and 13 proteins, respectively, which were significantly upregulated in the patients with severe pain one year after disc herniation. On the top of the list ranked by estimated increase we found C-X-C motif chemokine 5 (CXCM5; 217% increase), epidermal growth factor (EGF; 142% increase), and monocyte chemotactic protein 4 (MCP-4; 70% increase). Moreover, a clear overall difference in the serum cytokine profile between the chronic and the recovered patients was demonstrated. Thus, the present results may be important for future protein serum profiling of lumbar radicular pain patients with regard to prognosis and choice of treatment. We conclude that serum proteins may be measurable molecular markers of persistent pain after disc herniation.

## 1. Introduction

Lumbar radicular pain, also referred to as “sciatica,” is characterized by radiating pain that typically follows the dermatome of the affected nerve-root. When strict criteria are used, the prevalence rate is approximately 2% [1, 2]. Roughly 90% of the lumbar radicular pain cases are caused by intervertebral disc herniation with nerve-root compression [3]. Although a spontaneous recovery is seen in most of these patients, only 30% are completely pain-free one year after onset [4]. At present, there are no measurable molecular markers for patient prognosis, choice of treatment, or development of new compounds for persistent pain after disc herniation.

Earlier studies suggest that leakage of nucleus pulposus into the spinal canal after disc herniation may initiate immunological and inflammatory responses close to the nerve-roots [5, 6] that increase the activity in nociceptive pathways [7–11]. This inflammatory influence has been attributed to an upregulation of interleukins (ILs), tumor necrosis factor (TNF), matrix metalloproteinases (MMPs), nitric oxide (NO), and prostaglandins (PGs) in or around the herniated disc [12–14].

In addition, recent data point to increased levels of circulating cytokines in patients with chronic pain. For example, higher systemic IL-2 and TNF levels have been observed in patients with painful neuropathies compared to painless neuropathies [15]. Moreover, IL-6 and IL-8 appear to be elevated in serum of patients with radicular pain due to disc herniation [16, 17]. Thus, earlier observations suggest that disc herniation may be associated with an upregulation of several inflammatory molecules. In these previous studies, however, the methodological hindrance has limited the number of markers to a few proteins.

In the present study, in contrast, we take advantage of the new multiplex proximity extension assay (PEA) technology to simultaneously analyze the levels of 92 inflammatory proteins [18]. To adjust for multiplicity, we used the false discovery rate (FDR) approach in the statistical analysis of the data [19]. The aim of the present study was to investigate if it is possible to define an inflammatory fingerprint in the serum of patients with persistent lumbar radicular pain after disc herniation. Our results indicate that the PEA technology may be important for future protein serum profiling of lumbar radicular pain patients with regard to prognosis and choice of treatment.

## 2. Materials and Methods

### 2.1. Patients

Patients with lumbar radicular pain were recruited from the outpatient clinic at Oslo University Hospital, Ullevål, Norway, during the period 2007–2009. The inclusion criteria were age between 18 and 60 years, lumbar disc herniation confirmed by magnetic resonance imaging (MRI) with corresponding radicular pain, and positive Straight Leg Raising (SLR). The exclusion criteria were lumbar spinal stenosis, previous surgery for herniated disc at the same level or fusion at any level in lumbar spine, generalized musculoskeletal pain, inflammatory rheumatic disease, diabetic polyneuropathy, cardiovascular disease (NYHA III and IV), cancer, psychiatric disease, cauda equine syndrome, alcohol or drug abuse, recent surgery (within one month), pregnancy, poor Norwegian language, or non-European-Caucasian ethnicity. In total, 122 patients (82%) of the 148 patients who met the eligibility requirement were included in the intended follow-up assessment.

Surgical treatment was given to patients with persistent radicular pain lasting for more than 8 weeks, neurological deficits (sensory changes, muscle weakness, and depressed or absent deep tendon reflexes), and corresponding magnetic resonance imaging findings in the anticipated location. Patients who did not clearly fulfill these criteria were managed conservatively by a treatment comprised of a brief cognitive intervention, activity guidance during the acute phase of disc herniation, and, for the majority of patients, physiotherapy.

At inclusion, at 6 weeks and at 12-month follow-up, the patients were asked to rate their pain intensity in activity during the last week on a 10 cm visual analog scale (VAS) with endpoints “no pain” and “worst possible pain.” Hopkins symptom checklist (HSCL), a 25-item questionnaire, was used to register psychological distress, that is, anxiety and depression. The drop-out rate was 8% and ultimately, 112 patients were assessed at 12-month follow-up.

Among these 112 patients, patients with VAS > 6 at 12-month follow-up were defined as a high pain group (*n* = 23). Moreover, a gender and age matched sample from the same 112 patients with VAS < 1 at 12-month follow-up was defined as a low pain group (*n* = 22). The serum levels in inflammatory-related proteins in the high pain group versus the low pain group were analyzed. All participants received written information and signed an informed consent form. The study was approved by the Norwegian Regional Committee for Medical Research Ethics and the Norwegian Social Science Data Services.

### 2.2. Blood Sampling

At 12-month follow-up, venous blood was collected and kept on ice for 45 minutes. After centrifugation at 2,000 g for 10 min at 4°C, the supernatant serum was collected and stored in aliquots at −80°C until further analysis. Serum was used to analyze the biomarkers for the levels of 92 inflammatory proteins.

### 2.3. Proximal Extension Assay

In the present study we took advantage of the multiplex proximity extension assay (PEA) technology in which a panel of 92 proteins (see Supplementary Table 1 in Supplementary Material available online at http://dx.doi.org/10.1155/2016/3874964) is simultaneously analyzed [18]. The serum samples were assessed with Proseek Multiplex Inflammation I (Olink Bioscience, Uppsala, Sweden) using the PEA according to the manufacturer's instructions. Briefly, 3 *μ*L incubation mix containing two PEA probes, that is, antibodies equipped with single strand DNA oligonucleotide, against each protein was mixed with 1 *μ*L serum and the mixture was incubated at 8°C overnight. The mixture was then mixed with 96 *μ*L extension mix containing PEA enzyme and PCR reagents and incubated for 5 min at room temperature before the plate was transferred to a thermal cycler for 17 cycles of DNA amplification. A 96.96 Dynamic Array IFC (Fluidigm, CA, USA) was prepared and primed according to the manufacturer's instructions. 2.8 *μ*L of sample mixture was mixed with 7.2 *μ*L detection mix in a new 96-well plate and 5 *μ*L was loaded into the right side of the primed 96.96 Dynamic Array IFC. The unique primer pairs for each cytokine were loaded into the left side of the 96.96 Dynamic Array IFC, and the protein expression program was run in Fluidigm Biomark reader according to the instructions for Proseek.

### 2.4. Statistics

For the baseline characteristics, the Kolmogorov-Smirnov test was applied to test for normality. Unpaired Student's *t*-test was chosen to compare mean values for normally distributed data, whereas for nonnormally distributed data the mean values were compared by a nonparametric two-sided Mann-Whitney *U* test. Pearson Chi-square test was used to analyze the frequency of baseline categorical variables between the high and low pain group.

In order to identify differentially expressed serum proteins between the two groups of patients, protein-level analyses for each individual biomarker were performed. For a group of patients, the protein expression levels of some proteins were below the laboratory's limit of detection (LoD). Therefore, we used a multiple linear regression model with LoD imputed values in place of below-LoD values to test the differences between the groups.

Because of the difficulties associated with analyzing data with a high proportion of below-LoD values [20–22], only proteins for which the proportion of below-LoD values was less than 20% were included in the analyses. The false discovery rate (FDR) was controlled using the procedure of Benjamini and Hochberg (1995) [19]. The difference in expression levels was estimated by computing the ratio of the group medians on the original non-log scale.

The linear discriminant analysis [23, 24] was performed to visualize the differences between the two groups. Using vector notation and letting **x**_*ij*_ denote the vector of protein measurements of patient *j* in group *i* (with *i* = 1 corresponding to high pain and *i* = 2 corresponding to low pain), ${\overset{-}{x}}_{i}$ denote the sample mean vector in group *i*, and *n*_*i*_ denote the number of patients in group *i*, the discriminant score for each individual was computed as

(1) x−1−x−2′∑i=12∑j=1nixij−x−ixij−x−i′−1·xij−12x−1+x−2.

This resulted in a score, essentially based on a weighted average of the differentially expressed proteins, such that positive values indicated high levels of inflammatory proteins and negative values indicated low levels of inflammatory proteins.

The weighted protein score was visualized in a simple dot plot where each patient was represented by one dot. A positive score indicated a high inflammatory activity, whereas a negative score indicated a low inflammatory activity.

## 3. Results

**Figure 1:**
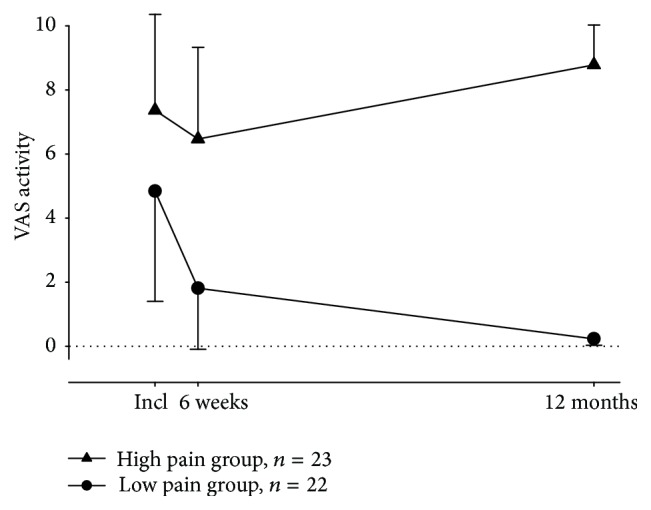
Intensity of pain in 45 disc herniation patients recruited from Oslo University Hospital, Ullevål, Norway. The patients were divided into two groups based on their clinical outcome measured by VAS at 12-month follow-up. The data are shown as mean ± SD.

The high pain group had more pain when they arrived at the hospital, at 6 weeks and 12-month follow-up, than the low pain group (Figure 1). More smoking and higher HSCL score were also observed in the high pain group (Table 1). Therefore, smoking and HSCL were corrected for in the further analyses, that is, in the multiple linear regression model to test the differences between the high and low pain group.

The analyses of the levels of 92 proteins showed 76 proteins for which at most 20% of the patients had expression levels below the LoD. Among these 76 proteins, we identified 41 with FDR < 0.10 and 13 with FDR < 0.05, which were considered upregulated in the patients with severe pain one year after disc herniation (Table 2). The analyses of the inflammation-related protein levels showed increased expression of many proteins in the high pain group compared to the low pain group.

On the top of the list ranked by estimated increase we found C-X-C motif chemokine 5 (CXCM5; 217% increase), epidermal growth factor (EGF; 142% increase), and monocyte chemotactic protein 4 (MCP-4; 70% increase).

**Figure 2:**
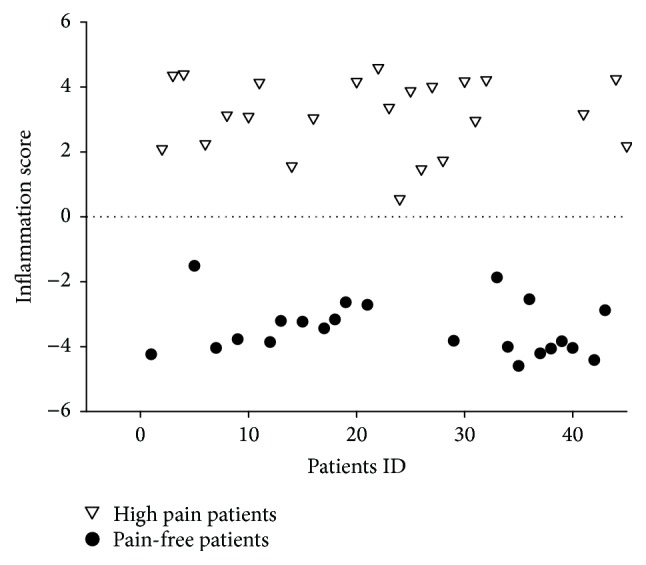
Linear discriminant analysis. An inflammation score was computed for each patient as a weighted average of the protein expression levels for the 41 biomarkers (FDR < 0.10). The scores were adjusted so that positive values indicate an ongoing inflammation and negative values indicate lack of inflammation. The scores are plotted for all patients (patient ID 1–45) in the study.

**Figure 3:**
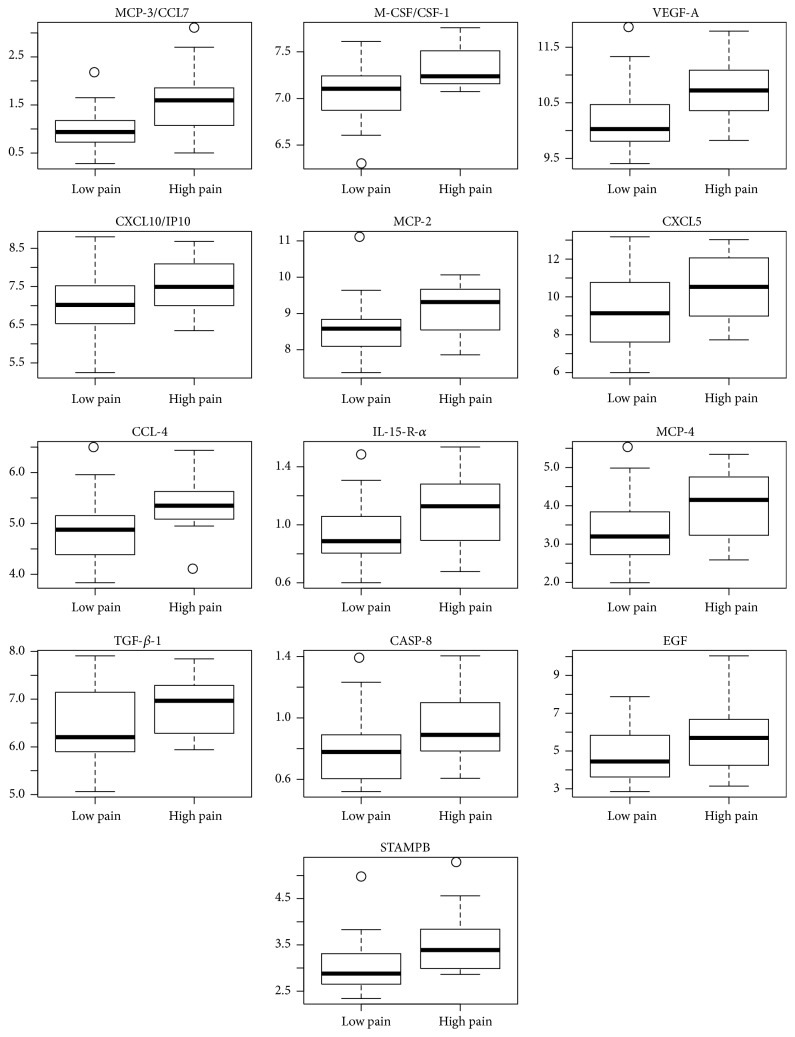
The expression of the 13 most significant proteins (FDR < 0.05). All 45 patients were included. The data are shown by box plot; median and IQR ± min/max.

For each patient, an inflammation score, that is, weighted average of the 41 protein levels, was also constructed. Notably, the two groups were completely separated; all high pain patients had positive scores, whereas all low pain patients had negative scores (Figure 2). Thus, an inflammatory fingerprint in the serum of the patients with persistent lumbar radicular pain after disc herniation was demonstrated. The data showed a clear increase in cytokine levels in the patients that developed chronic pain (Figure 3).

## 4. Discussion

In the present study, we examined the serum levels of 92 inflammatory proteins in patients one year after lumbar disc herniation. Among these, 41 proteins with FDR < 0.10, including IL-6 and IL-8, seemed to be upregulated in the chronic pain patients compared to the patients that fully recovered within one year. Hence, in accordance with our earlier ELISA findings from the same cohort where IL-6 and IL-8 in almost all patients were increased the first weeks after disc herniation [17, 25], the present data showed that patients that develop long lasting radicular pain may be different from patients that recover with regard to measurable cytokines in serum. Moreover, a clear overall difference in the serum cytokine profile between the chronic and the recovered patients was demonstrated.

Many of the upregulated proteins with FDR < 0.05 were chemokines and other molecules associated with inflammation, activation of macrophages, and/or immune responses including regulators of glial cells. For example, C-X-C motif chemokine 5 (CXCL5) is a neutrophil chemoattractant which increases the expression of several proinflammatory mediators [26]. Moreover, monocyte chemotactic proteins 2, 3, and 4 (MCP-2, MCP-3/CCL7, and MCP-4) recruit macrophages [27], whereas C-X-C motif chemokine 10 (CXCL10/IP10) activates T-cells [28] and microglia [29]. Macrophages and microglia cells may also be activated by macrophage colony stimulating factor (M-CSF/CSF-1) thought to be upregulated in nucleus pulposus tissue after disc herniation [10, 30].

Several of the upregulated proteins with FDR < 0.05 have also previously been linked to healing and tissue regeneration. C-C motif chemokine 4 (CCL4) that reduces the expression of TNF and IL-1*β* [31] may be involved in nucleus pulposus resorption after disc herniation [32]. Vascular endothelial growth factor A (VEGF-A) supports healing, regeneration, and revascularization [33–35] and facilitates opening of the blood-nerve-barrier [36]. Interleukin-15 receptor-*α* (IL-15R*α*) is expressed in monocytes, NK cells, and T-cells [37].

In addition, some of the upregulated proteins with FDR < 0.05 may induce cell differentiation, apoptosis, and neuronal loss and influence cell cycle regulation. Epidermal growth factor (EGF) stimulates cell growth, proliferation, and differentiation [38]. Moreover, transforming growth factor *β* (TGF*β*) has important immune-regulative role and may reverse inflammation [39], whereas caspase-8 (CASP-8) is involved in FAS mediated apoptosis [40, 41] and cleavage of cytoskeletal proteins. STAM-binding protein (STAMBP) is a zinc metalloprotease involved in cell cycle regulation recently linked to the neuronal loss underlying microcephaly-capillary malformation syndrome [42].

### 4.1. Methodological Considerations

In PEA, each target molecule is recognized by a pair of affinity binders in which both have to bind the same target molecule prior to signal amplification via qPCR. Similar to other proximity assays [43, 44], the PEA technology also has an increased assay specificity. The specificity of the assay has been validated using a complete pool of PEA probes to analyze either all or submixes of eight antigens, for which significant signal over background was obtained only when the cognate antigen was present [18]. As previously described, the PEA technology has a sensitivity in low pM range [18].

Still, as some of the protein expressions were below the LoD, the actual expression levels of these proteins remain unknown. As high proportions of below-LoD measurements lead to tests with low power, we opted to only analyze the proteins for which the proportion of below-LoD measurements was at most 20%. Moreover, the two groups were balanced in terms of age, sex, and treatment but differed in smoking habits and HSCL scores (Table 1). Our linear regression model was therefore adjusted for smoking and HSCL scores to exclude the possibility that any differences found actually were due to differences in smoking and HSCL.

In the present study, we examined 92 serum proteins. Thus, the analyses of the data include multiple testing, that is, associated with many challenges. Clearly, we cannot control the family wise error rate by Bonferroni correction as such corrections tend to be far too conservative, resulting in statistical procedures with very low power. However, since this is a study where the focus is on evaluation of the methodology (proof of concept), a limited rate of false positives is acceptable. We therefore controlled the false discovery rate using the procedure of Benjamini and Hochberg [19].

Given a FDR of 0.10 and 0.05, up to 1/10 and 1/20 of our findings are likely to be false positives. Importantly, however, almost all inflammatory markers moved in the same direction. Moreover, although a few false positive markers may be present, our study clearly demonstrated that inflammatory profiling can be used to characterize chronic sciatica patients. The present data also showed that the PEA is a reliable and sensitive method to study proteins in serum associated with clinical measures.

Despite the above mentioned challenges, the linear discriminant analysis demonstrated a clear difference between the high pain group and the low pain group. A weighted average of the differentially expressed proteins with FDR < 0.10 was used [24]. This allowed us to summarize our multivariate data in a single objective measure [19]. Notably, the two groups were completely separated; all high pain patients had positive scores, whereas all low pain patients had negative scores. Thus, even though there might be one or two false positive markers, an inflammatory fingerprint in the serum of the patients that developed persistent lumbar radicular pain after disc herniation was defined by the PEA technology.

### 4.2. Study Limitations

Although the present study had a prospective design when it comes to the pain ratings, the serum protein levels of the patients were only measured at one time point, that is, 12 months after disc herniation. Thus, it is hard to tell whether the observed differences in serum proteins are a trait or a state of the patients that develop chronic pain. Moreover, medication and other life style factors may also influence the serum protein levels. However, the analgesics and anti-inflammatory-drugs taken by the patients were higher in the high pain group than in the low pain group. Therefore, it seems likely that the effect of medication reduces rather than increases the difference in inflammatory serum proteins. Nevertheless, the results demonstrated that it is possible to detect differences between persistent and nonpersistent pain patients. The fact that this protein pattern can be detected in serum suggests that the PEA technology may have a clinical potential.

## 5. Conclusion

In summary, the present data demonstrated that sciatica patients with a chronic outcome have increased levels of inflammatory proteins in serum one year after disc herniation. This is to our knowledge the first protein serum profiling study of patients with chronic lumbar radicular pain. An inflammatory PEA-based fingerprint of these persistent pain patients was demonstrated. We conclude that serum proteins may be measurable molecular markers of patients that develop persistent pain after disc herniation. The pathophysiological relevance of these proteins, however, remains to be investigated.

## Supplementary Material

In the Proximity Extension Assay (PEA) each of the 92 human protein biomarkers were addressed by a pair of oligonucleotide-labeled antibodies. The amount of the proteins was measured by quantitative real-time PCR. The dual recognition DNA-coupled method excluded cross-reactivity in the detection process.

## Figures and Tables

**Table 1:** Baseline characteristics of patients grouped in the high and low pain group.

	Low pain	High pain	*p* value
Gender, men/women (%)	11/11/(50/50)	12/11 (52/48)	0.884^a^
Age, mean (SD)	40 (9)	41 (12)	0.697^b^
Current smoker, yes/no (%)	6/16 (27/73)	13/10 (57/43)	0.047^a^
Treatment, surgery/conservative (%)	10/12 (45/55)	8/15 (35/65)	0.465^a^
HSCL total score, mean (SD)	1.53 (0.41)	2.09 (0.56)	0.001^c^

^a^Pearson Chi-square, ^b^unpaired Student's *t*-test, and ^c^Mann-Whitney *U* test. SD: standard deviation, and HSCL: Hopkins symptom checklist.

**Table 2:** List of biomarkers which are significantly differentially expressed when the false discovery rate (FDR) is 0.10 and 0.05. The rightmost column shows the estimated increase on the original non-log scale.

Biomarker	*p* value	FDR adjusted *p* value	Estimated increase		
MCP-3/CCL7	0.0006	0.047	50.6%	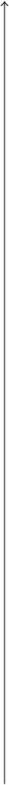	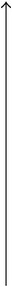
M-CSF/CSF-1	0.001	0.047	20.2%		
VEGF-A	0.0015	0.047	47.6%		
CXCL10/IP10	0.0017	0.047	55.2%		
MCP-2	0.0023	0.047	56.6%		
CXCL5	0.0024	0.047	217.0%		
CCL-4	0.0034	0.047	43.4%		
IL-15-R-alpha	0.0036	0.047	15.2%		
MCP-4	0.004	0.047	69.9%		
TGF-beta-1	0.0041	0.047	52.3%		
CASP-8	0.0051	0.047	14.3%		
EGF	0.0064	0.047	142.0%		
STAMPB	0.0071	0.047	40.4%		FDR < 0.05
				
IFNg	0.0102	0.0553	25.0%		
IL-6	0.0118	0.0596	68.5%		
TRAIL	0.0119	0.0596	16.6%		
LIGHT-TNFSF14	0.0147	0.0656	35.6%		
CX3CL1	0.0147	0.0656	18.5%		
CXCL6	0.0153	0.0656	82.4%		
CXCL9-MIG	0.0165	0.0656	39.7%		
SIRT2	0.0173	0.0656	44.9%		
IL-10-R-beta	0.0179	0.0656	19.6%		
MCP-1-CCL2	0.0191	0.0656	36.2%		
HGF	0.0195	0.0656	37.9%		
Eotaxin-1	0.0198	0.0656	31.3%		
AXIN1	0.021	0.0656	11.2%		
CCL19	0.0217	0.0656	44.0%		
CDCP1	0.0223	0.0656	26.7%		
CD40	0.0233	0.0656	18.8%		
IL-8	0.0238	0.0656	47.5%		
SULT1A1	0.0256	0.0656	21.0%		
Beta-NGF	0.0286	0.0679	11.8%		
OSM	0.0298	0.0685	78.7%		
CCL20	0.0298	0.0685	43.7%		
TWEAK-TNFSF12	0.0316	0.0687	21.7%		
EIF4EBP1	0.0339	0.0716	59.2%		
MIP-1-alpha-CCL3	0.0384	0.0788	16.1%		
CXCL11	0.0384	0.0788	54.6%		
IL12B	0.0386	0.0788	28.9%		
IL18R1	0.0436	0.0828	20.8%		
CXCL1	0.0459	0.0852	52.8%	FDR < 0.10	
