# Supplementary material for: Inflammatory Serum Protein Profiling of Patients with Lumbar Radicular Pain One Year after Disc Herniation
Source: Int J Inflam. 2016 May 11;2016:3874964. doi: 10.1155/2016/3874964 (PMC4879232; doi:10.1155/2016/3874964)
Supplement: Supplementary file 1 — In the Proximity Extension Assay (PEA) each of the 92 human protein biomarkers were addressed by a pair of oligonucleotide-labeled antibodies. The amount of the proteins was measured by quantitative real-time PCR. The dual recognition DNA-coupled method excluded cross-reactivity in the detection process. [file 3874964.f1.pdf]

Supplementary Table 1

List of the proteins included in this study (Source: www.olinke.com)

Long name (short name)	Uniprot	LoD (pg/ml)*
Adenosine Deaminase (ADA)	P00813	0.48
Artemin (ARTN)	Q5T4W7	0.24
Axin-1 (AXIN1)	O15169	61
Beta-nerve growth factor (Beta-NGF)	P01138	0.48
Brain-derived neurotrophic factor (BDNF)	P23560	–
Caspase 8 (CASP-8)	Q14790	0.48
C-C motif chemokine 4 (CCL4)	P13236	1.9
C-C motif chemokine 19 (CCL19)	Q99731	15
C-C motif chemokine 20 (CCL20)	P78556	7.6
C-C motif chemokine 23 (CCL23)	P55773	31
C-C motif chemokine 25 (CCL25)	O15444	3.8
C-C motif chemokine 28 (CCL28)	Q9NRJ3	61
CD40L receptor (CD40)	P25942	0.01
CUB domain-containing protein 1 (CDCP1)	Q9H5V8	0.12
C-X-C motif chemokine 1 (CXCL1)	P09341	3.8
C-X-C motif chemokine 5 (CXCL5)	P42830	0.95
C-X-C motif chemokine 6 (CXCL6)	P80162	7.6
C-X-C motif chemokine 9 (CXCL9)	Q07325	0.95
C-X-C motif chemokine 10 (CXCL10)	P02778	7.6
C-X-C motif chemokine 11 (CXCL11)	O14625	7.6
Cystatin D (CST5)	P28325	1.9
Delta and Notch-like epidermal growth factor-related receptor (DNER)	Q8NFT8	0.95
Eotaxin-1 (CCL11)	P51671	3.8
Eukaryotic translation initiation factor 4E-binding protein 1 (4E-BP1)	Q13541	–
Fibroblast growth factor 5 (FGF-5)	Q8NF90	1.9
Fibroblast growth factor 19 (FGF-19)	O95750	7.6
Fibroblast growth factor 21 (FGF-21)	Q9NSA1	31
Fibroblast growth factor 23 (FGF-23)	Q9GZV9	122
Fms-related tyrosine kinase 3 ligand (Flt3L)	P49771	0.01
Fractalkine (CX3CL1)	P78423	15.3
Glial cell line-derived neurotrophic factor (hGDNF)	P39905	0.01
Hepatocyte growth factor (HGF)	P14210	7.6
Interferon gamma (IFN-gamma)	P01579	15.3
Interleukin-1 alpha (IL-1 alpha)	P01583	0.48
Interleukin-2 (IL-2)	P60568	30.5
Interleukin-2 receptor subunit beta (IL-2RB)	P14784	15
Interleukin-4 (IL-4)	P05112	0.24
Interleukin-5 (IL-5)	P05113	3.8
Interleukin-6 (IL-6)	P05231	0.12
Interleukin-7 (IL-7)	P13232	0.24
Interleukin-8 (IL-8)	P10145	0.03
Interleukin-10 (IL-10)	P22301	0.48
Interleukin-10 receptor subunit alpha (IL-10RA)	Q13651	3.8
Interleukin-10 receptor subunit beta (IL-10RB)	Q08334	0.12
Interleukin-12 subunit beta (IL-12B)	P29460	0.12
Interleukin-13 (IL-13)	P35225	7.6
Interleukin-15 receptor subunit alpha (IL-15RA)	Q13261	0.95
Interleukin-17A (IL-17A)	Q16552	3.8
Interleukin-17C (IL-17C)	Q9P0M4	31
Interleukin-18 (IL-18)	Q14116	0.06
Interleukin-18 receptor 1 (IL-18R1)	Q13478	0.06
Interleukin-20 (IL-20)	Q9NYY1	7.6
Interleukin-20 receptor subunit alpha (IL-20RA)	Q9UHF4	1.9
Interleukin-22 receptor subunit alpha-1 (IL-22 RA1)	Q8N6P7	0.24
Interleukin-24 (IL-24)	Q13007	1.9
Interleukin-33 (IL-33)	O95760	3.8
Latency-associated peptide transforming growth factor beta 1 (LAP TGF-beta-1)	P01137	61
Leukemia inhibitory factor (LIF)	P15018	3.8
Leukemia inhibitory factor receptor (LIF-R)	P42702	30.5
Macrophage colony-stimulating factor 1 (CSF-1)	P09603	0.004
Macrophage inflammatory protein 1-alpha (MIP-1 alpha)	P10147	0.06
Matrix metalloproteinase-1 (MMP-1)	P03956	1.9
Matrix metalloproteinase-10 (MMP-10)	P09238	0.95
Monocyte chemotactic protein 1 (MCP-1)	P13500	0.03
Monocyte chemotactic protein 2 (MCP-2)	P80075	0.06
Monocyte chemotactic protein 3 (MCP-3)	P80098	0.48
Monocyte chemotactic protein 4 (MCP-4)	Q99616	7.6
Natural killer cell receptor 2B4 (CD244)	Q9BZW8	0.06
Neurotrophin-3 (NT-3)	P20783	0.12
Neurturin (NRTN)	Q99748	3.8
Oncostatin-M (OSM)	P13725	0.03
Osteoprotegerin (OPG)	O00300	0.24
Programmed cell death 1 ligand 1 (PD-L1)	Q9NZQ7	3.8
Protein S100-A12 (EN-RAGE)	P80511	122
Signaling lymphocytic activation molecule (SLAMF1)	Q13291	31
SIR2-like protein 2 (SIRT2)	Q8IXJ6	7.6
STAM-binding protein (STAMPB)	O95630	7.6
Stem cell factor (SCF)	P21583	1.9
Sulfotransferase 1A1 (ST1A1)	P50225	244
T cell surface glycoprotein CD6 isoform (CD6)	Q8WWJ7	0.06
T-cell surface glycoprotein CD5 (CD5)	P06127	0.06
Thymic stromal lymphopoietin (TSLP)	Q969D9	3.8
TNF-beta (TNFB)	P01374	0.24
TNF-related activation-induced cytokine (TRANCE)	O14788	3.8
TNF-related apoptosis-inducing ligand (TRAIL)	P50591	0.95
Transforming growth factor alpha (TGF-alpha)	P01135	0.48
Tumor necrosis factor (Ligand) superfamily, member 12 (TWEAK)	Q4ACW9	1.9
Tumor necrosis factor (TNF)	P01375	0.48
Tumor necrosis factor ligand superfamily member 14 (TNFSF14)	O43557	0.95
Tumor necrosis factor receptor superfamily member 9 (TNFRSF9)	Q07011	0.03
Urokinase-type plasminogen activator (uPA)	P00749	0.12
Vascular endothelial growth factor A (VEGF-A)	P15692	0.06

*Limit of detection (LoD) defined as 3 standard deviations above background
